# A simple and low-cost approach for irreversible bonding of polymethylmethacrylate and polydimethylsiloxane at room temperature for high-pressure hybrid microfluidics

**DOI:** 10.1038/s41598-021-83011-8

**Published:** 2021-03-01

**Authors:** Sara Hassanpour-Tamrin, Amir Sanati-Nezhad, Arindom Sen

**Affiliations:** 1 Pharmaceutical Production Research Facility, Department of Chemical and Petroleum Engineering, Schulich School of Engineering, University of Calgary, 2500 University Drive N.W., Calgary, AB T2N 1N4 Canada; 2 Biomedical Engineering Graduate Program, University of Calgary, 2500 University Drive N.W., Calgary, AB T2N 1N4 Canada; 3 BioMEMS and Bioinspired Microfluidic Laboratory, Department of Mechanical and Manufacturing Engineering, Schulich School of Engineering, University of Calgary, 2500 University Drive N.W., Calgary, AB T2N 1N4 Canada; 4 Center for Bioengineering Research and Education, Schulich School of Engineering, University of Calgary, 2500 University Drive N.W., Calgary, AB T2N 1N4 Canada

**Keywords:** Biomedical engineering, Lab-on-a-chip, Microfluidics

## Abstract

Microfluidic devices have been used progressively in biomedical research due to the advantages they offer, such as relatively low-cost, rapid and precise processing, and an ability to support highly automated analyses. Polydimethylsiloxane (PDMS) and polymethylmethacrylate (PMMA) are both biocompatible materials widely used in microfluidics due to their desirable characteristics. It is recognized that combining these two particular materials in a single microfluidic device would enable the development of an increasingly in-demand array of new applications, including those requiring high flow rates and elevated pressures. Whereas complicated and time-consuming efforts have been reported for bonding these two materials, the robust adhesion of PDMS and PMMA has not yet been accomplished, and remains a challenge. In this study, a new, simple, efficient, and low-cost method has been developed to mediate a strong bond between PMMA and PDMS layers at room temperature in less than 5 min using biocompatible adhesive tape and oxygen plasma treatment. The PDMS–PMMA bond was hydrolytically stable, and could tolerate a high influx of fluid without any leakage. This study addresses the limitations of existing approaches to bond these materials, and will enable the development of highly sought high-pressure and high-throughput biomedical applications.

## Introduction

Microfluidic technology emerged in the early 1990s and has since attracted considerable interest for efficient fluid processing, manipulation and control^1,2^. Compared to more traditional methods, microfluidic systems require small sample volumes, thereby minimizing reagent consumption and analysis time as well as increasing automation capabilities^3,4^. The progressive utilization of microfluidic devices is very evident in biological and medical research, where it has supported more powerful analyses and enabled faster, cheaper and more accessible diagnostics.

The materials used to initially manufacture microfluidic devices were silicon and glass^5^. Whereas these both still find use in electrophoretic and solvent-based applications, advances in microdevice fabrication technologies have now enabled a much wider range of materials to be used^6^. Polymers, due to their simple and low-cost advantages, have become viable alternatives to silicon and glass for fabrication of these systems^7,8^. Two of the most widely used polymers for the production of microfluidic devices in biomedical research are polydimethylsiloxane (PDMS) and polymethylmethacrylate (PMMA), due to their biocompatibility, cost-effectiveness and ease of fabrication^7,8^. PDMS is a silicon-based elastomer^9,10^ with high gas permeability, flexibility, and optical transmissivity which makes it an ideal choice for bio-based microfluidic applications such as mimicking the cellular environment for culturing and experimenting with cells^11^. However, challenges associated with PDMS include channel deformation, sample absorption, and low solvent resistivity which are limiting factors in many bioanalytical microfluidic applications^4,11,12^. By contrast, PMMA is an acrylic-based thermoplastic^13^ exhibiting good mechanical stability, chemical (acid and base) resistivity, and high transparency^14,15^. The refractive index of PMMA (1.49) indicates glass-like optical clarity and transparency^16^. Notably, compared to the glass, PMMA is inexpensive and features lightweight and superior toughness while offering easier and more cost-effective fabrication^17,18^.

Whereas PDMS is highly biocompatible and suitable for in vivo and in vitro biomedical models, its use in high-throughput and lengthy biomedical applications is limited due to its tendency for non-specific molecule adsorption and the release of small non-crosslinked PDMS molecules. Microchannels made of PDMS can absorb organic solvents and proteins, leading to clogging or even cell adhesion problems. Microchannels manufactured from PMMA do not suffer from these limitations. However, PMMA is not suitable for the fabrication of integrated flexible elements, such as porous membranes for cell culture^19,20^, and conductive and stretchable microfluidic sensors/electrodes^21,22^, for which PDMS has been shown to be highly effective. Strategically combining elastomeric PDMS and thermoplastic PMMA into a single hybrid device would enable the advantages of each material to be realized while simultaneously minimizing their limitations, thereby enabling a new range of microfluidic applications^15,23–27^. For instance, membrane-based micropumps or microvalves are microfluidic devices which benefit from the combination of thermoplastic and elastomeric materials^28,29^.

Whereas hybrid microfluidic devices composed of elastomeric and thermoplastic materials have been widely reported in medical and industrial applications, bonding between such materials is challenging due to their different physicochemical properties^7,8,25–27,29^. Table 1 shows a summary of the techniques previously reported for bonding of PDMS to PMMA. Generally, attempts to bond together PDMS and PMMA have involved either surface modification (i.e. direct bonding with no additional material at the interface), or the addition of an adhesive at the interface (indirect bonding)^30–34^. Surface modification using oxygen plasma treatment increases surface energy and has been shown to promote the irreversible bonding of PDMS to PDMS or to glass substrates via the interaction of functionalized silanol groups (SiOH) which lead to the formation of strong Si–O–Si bonds^27,30,35^. However, PDMS and PMMA cannot be directly bonded in this manner as they only exhibit weak van der Waals interactions after oxygen plasma treatment^27^, unless the PMMA surface is chemically modified. For example, PMMA substrates modified with 3-aminopropyltriethoxysilane (APTES)^31,34^ and tetraethyl orthosilicate (TEOS)^30^ exhibit upregulated bonding to PDMS following oxygen plasma exposure. However, techniques based on chemical modifications not only need to be optimized for surface treatment conditions, but also require relatively high temperature and pressure to realize bonding which can contribute to microchannel clogging and deformation^30,31,34^.

**Table 1:** Comparison of various methods developed for bonding PMMA and PDMS layers.

Bonding method	Applied pressure	Temperature (°C)	Time required (min)	Tensile strength (psi)	Burst pressure (psi)	Leakage resistance (mL min^−1^)	Refs.
Modification of PMMA with oxygen plasma and 3-aminopropyltriethoxysilane (APTES), followed by corona discharge treatment of PMMA and PDMS layers	Not reported	65	> 120	~ 363	> 45	Not reported	^34^
Oxygen plasma treatment of PMMA and PDMS, followed by surface modification of PMMA with APTES	Not reported	Room temperature	> 15	~ 56	~ 76.5	60	^25^
Chemical Gluing, formation of amine–epoxy bond at the interface of PMMA and PDMS layers	Not reported	Room temperature	60	~ 26	~ 74	30	^26^
Placing a thin, uncured PDMS layer between PMMA and PDMS layers, followed by curing at 90 °C for 3 h	50 kPa	90	> 180	~ 2	Not reported	0.8	^27^
Placing a thin PDMS (already coated onto the adhesive film and cured at 80 °C for one hour) between PMMA and PDMS layers, followed by oxygen plasma treatment of the substrates	Not reported	80	> 60	Not reported	Not reported	0.096	^29^
Oxygen plasma treatment of PMMA and PDMS, followed by surface modification with tetraethoxysilane (TEOS)	Clamped	50	60	Not reported	Not reported	Not reported	^30^
Chemical modification of PMMA with APTES, followed by oxygen plasma treatment of PMMA and PDMS layers	Pressed by 0.5 kg weight	80	60	~ 164	Not reported	Not reported	^31^
A thin silica coated on PMMA, followed by oxygen plasma treatment of PMMA and PDMS layers	Not reported	80	120	Not reported	~ 43.5	Not reported	^32,33^

Indirect bonding is an approach involving a liquid or solid adhesive as an intermediate layer at the interface between two different materials. The formation of a permanent bond between PMMA and PDMS using a liquid adhesive requires the application of an adhesive between those surfaces, and then hardening via processes such as chemical reaction, ultraviolet (UV) radiation, solvent evaporation, heating or cooling^36^. For example, Chow et al*.*^27^ placed a thin (10–25 µm), uncured liquid PDMS layer at the interface between solid PDMS and PMMA, and cured the resulting construct at 90 °C for three hours to achieve adhesion between the PDMS and PMMA substrates. In another study, a thin coating of silica gel on a PMMA substrate formed an intermediate layer when subjected to heat, which enabled adhesion with PDMS following exposure to oxygen plasma treatment^32,33^. However, in all these studies, reported low tensile strength and leakage indicate that the resulting bonds were not robust (see Table 1)^27,32,33^. Notably, the main factor that limits the use of liquid adhesives in microfluidic devices is their tendency to flow into, and clog, microchannels^27,37^. Moreover, the challenge of applying an intermediate layer uniformly, and significant bake times (2–3 h) at high temperatures (80–90 °C) serve as additional complexities in these techniques^32,33^. For these reasons, the use of liquid adhesives to bond PDMS and PMMA are no longer considered to be feasible for microfluidic applications.

Compared to liquid adhesives, solid adhesives exhibit a significantly reduced magnitude of microchannel clogging^38,39^. Pressure sensitive adhesive (PSA) tape is a solid adhesive film mainly composed of elastomers (rubbers, acrylates and silicones)^39,40^. Commercial PSA tapes (e.g. 3 M, Research Adhesive, ABI Tape, Coroplast, Berry, and Avery Dennison) are inexpensive, simpler to use than liquid adhesives, and can reduce the time required to assemble a microfluidic device compared to some other bonding methods. Their use allows for an adhesive layer with uniform thickness at the interface between two substrates, and PSA has been shown not to deform and enter microchannels under high pressure or elevated temperatures, eliminating concerns around microchannel clogging^39^. Moreover, their use is readily scalable, thereby supporting the large-scale production of microfluidic devices. Importantly, they have demonstrated excellent biocompatibility, making them promising candidates for the fabrication of microfluidic channels in biomedical analysis devices. Kratz et al*.*^41^ investigated different types of PSA (three acrylic and one silicone-adhesive-based) for physical and optical properties, including biocompatibility. They examined detrimental effects of the adhesive layers on living cells using metabolic and live/dead bioassays and demonstrated their safety and feasibility for a number of applications, including cell-based microfluidic devices^41^. The biocompatibility of PSA makes it an excellent candidate to serve as an interfacial agent in the creation of hybrid PDMS–PMMA microfluidic devices for use in biomedical engineering applications.

Whereas the use of solid adhesive films including PSA has been revealed as a rapid and low-cost approach to fabricate PMMA microfluidic devices^38,39,42,43^, reports describing the use of PSA for the production of hybrid PDMS–PMMA microfluidic devices are very limited^29,32,33,39^. The main reason for this is that although PSA tape can firmly adhere to PMMA substrates simply by applying pressure at room temperature^38,39,42^, its ability to readily adhere to PDMS remains a significant challenge^39^. However, given the potential benefits of hybrid PDMS–PMMA microfluidic devices, there is significant motivation to establish new methods to permanently bond PSA to PDMS. Although PSA tape has been used to seal PDMS microfluidic devices, bond failure was evident at working pressures higher than 14 psi^44,45^, thereby precluding their use in high-pressure microfluidic applications like centrifugal microfluidic assays^46^. In one published study, Tan et al*.*^29^ used a thin layer of PDMS coated onto PSA tape at the interface between PMMA and PDMS. Creating a PMMA-PDMS construct required long bake times (> 1 h) at relatively high temperatures (80 °C) which elevated the probability of deformation and clogging in microchannels^29,44,45^. Despite these efforts, the resulting PDMS–PMMA device could only withstand a very low maximum flow rate of 0.096 mL min^−1^ (inadequate for high-throughput microfluidic applications), suggesting a lack of significant bonding between PDMS and PSA^29^.

In the current study, an adhesive-based technique was successfully developed to achieve a very strong bond between PMMA and PDMS using double-sided PSA tape. This novel method resulted in the creation of a robust bond with long term hydrolytic stability within 5 min at room temperature without the need for any chemical treatment. Given the required time for bonding (5 min), this method is significantly faster than other PDMS–PMMA bonding procedures reported to date (see Table 1). The quality of PDMS–PMMA bonding was characterized comprehensively using burst pressure, leakage and tensile bonding strength tests. The results obtained demonstrated that microfluidic devices manufactured by sandwiching a PSA interface between PDMS and PMMA using this new bonding approach have the integrity to withstand very high working pressures without exhibiting signs of leakage, microchannel clogging or microchannel deformation. The simple, reproducible and cost-effective nature of this approach lends itself to widespread adoption for the production of desirable hybrid PDMS–PMMA microfluidic devices such as microvalves and micropumps, as well as high pressure and high-throughput microfluidics including centrifugal microfluidic assays^46^ and liquid chromatographic separations^47,48^, which have numerous applications both within, and well beyond, biomedical engineering.

## Bonding strategy

**Figure 1:**
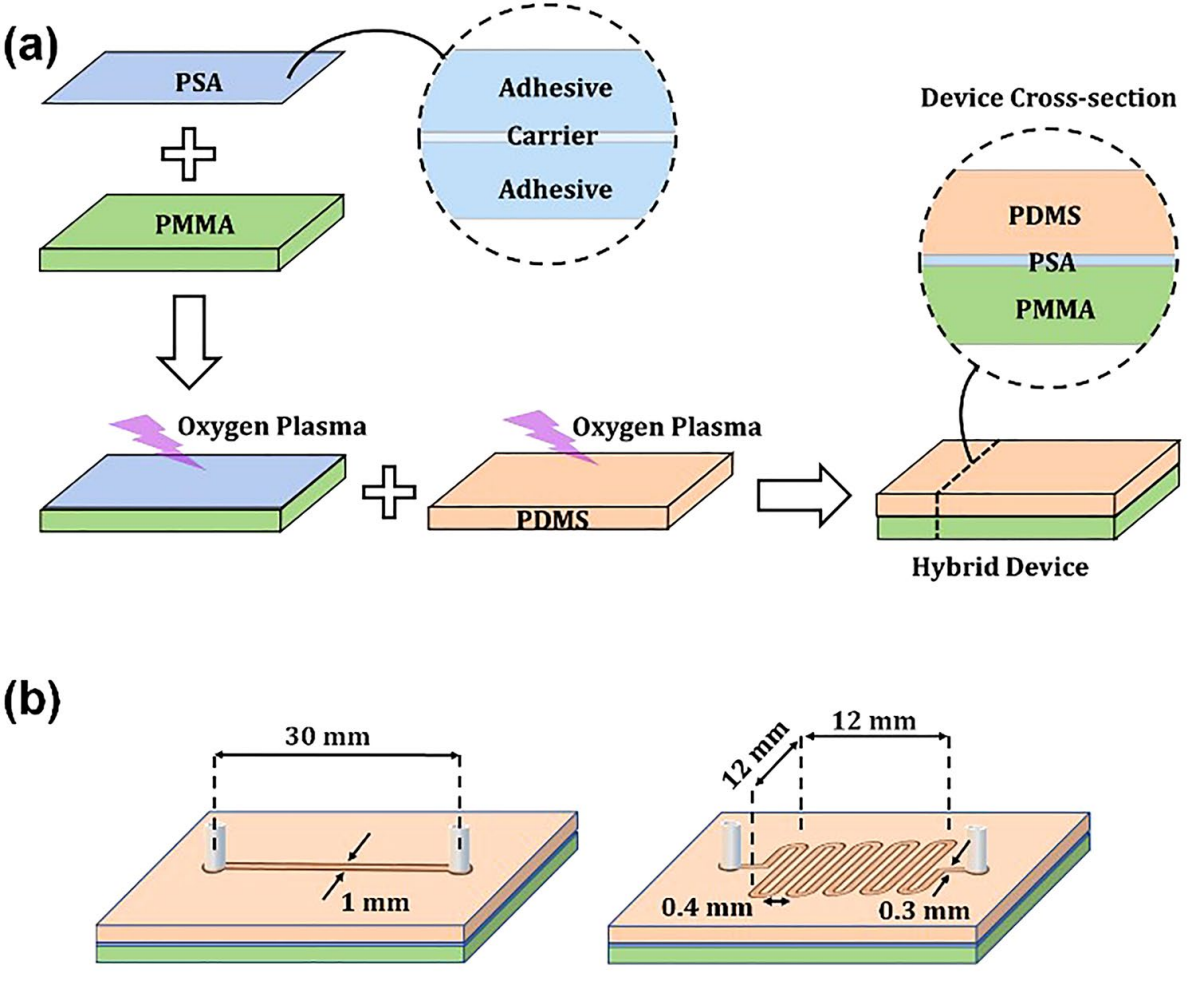
Bonding strategy and microchannel design. (**a**) Schematic of the method to bond PMMA and PDMS layers at room temperature (23 °C). This method was an adhesive-based technique involving oxygen plasma treatment of PDMS bonded to adhesive tape already laminated on PMMA. Double-sided PSA tape is an adhesive strip that is coated on both sides of a flexible plastic film as a carrier. (**b**) Two types of microfluidic chips were fabricated using the adhesive-based technique developed in this work; a simple straight microchannel (total length of 30 mm, height of 0.4 mm, and width of 1 mm) and a serpentine microchannel (total length of 190 mm, height of 0.4 mm, width of 0.3 mm, and gap size of 0.4 mm between the microchannels).

A schematic representation of the bonding process is shown in Fig. 1a. Double-sided PSA tape, a flexible plastic film (carrier) coated on each side by a strip of PSA, was used to facilitate the adhesion of two different materials^39,40^. The PMMA substrate was first gently cleaned using isopropanol solvent and wipes, followed by lamination of the PSA onto the PMMA substrate. The PMMA–PSA construct was compressed via a trigger clamp for 2 min to facilitate bonding. The PSA (already laminated on the PMMA) and PDMS surfaces were then exposed to oxygen plasma treatment (PE-25, Plasma-Etch, Inc.) for 1 min under the working condition of 15 W, 25 mL min^−1^ oxygen flow, and 700 mTorr chamber pressure to increase their surface energy in an attempt to facilitate the bonding of these two materials. Finally, the treated PDMS layer was placed on the PSA, and the entire construct was kept in conformal contact (slightly compressed between thumb and index fingers) at room temperature (23 °C) for 10–15 s.

## Result and discussion

### Surface characterization

**Figure 2:**
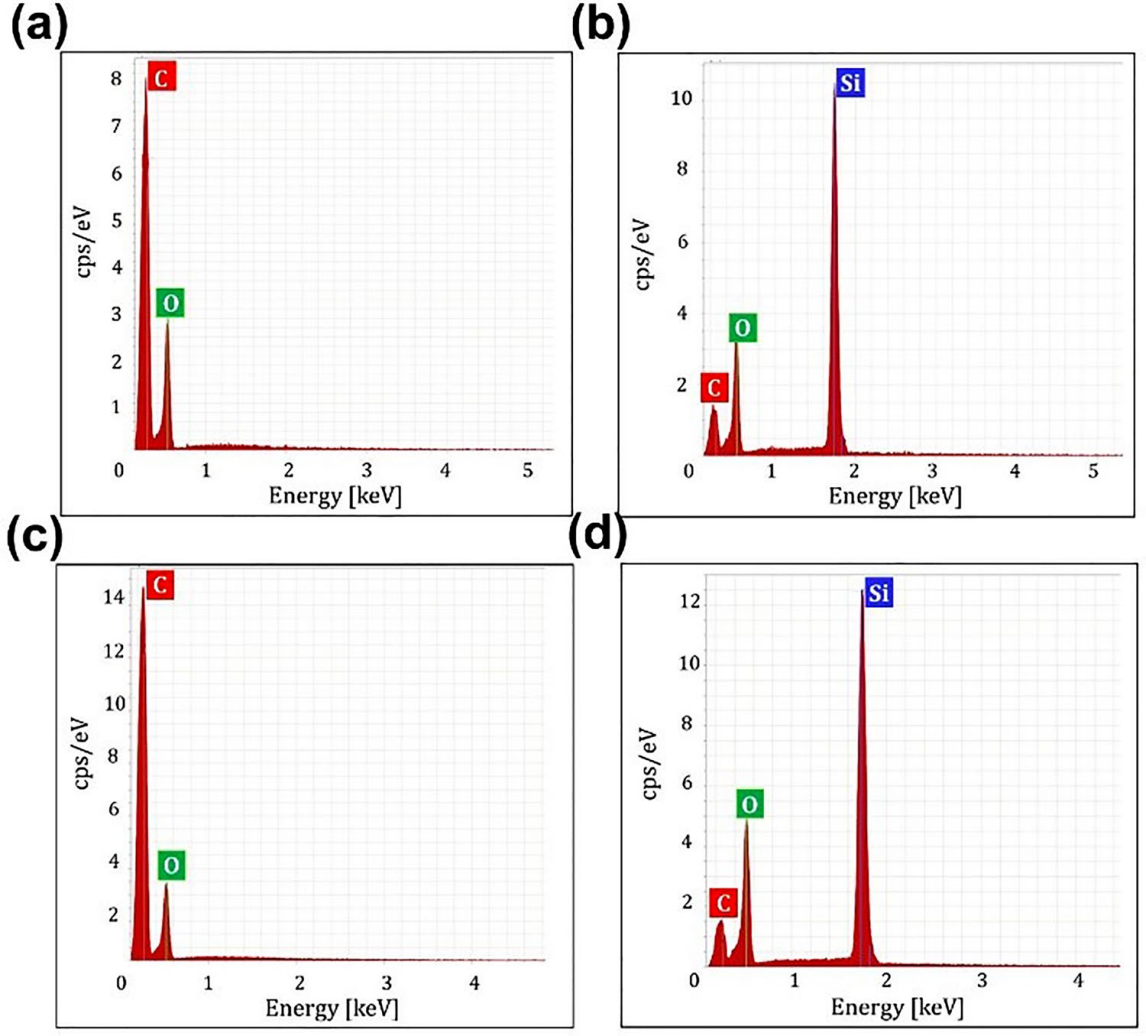
Energy-dispersive X-ray spectra of (**a**) PMMA, (**b**) PDMS, (**c**) acrylic-based PSA, and (**d**) silicon-based PSA. The presence of carbon (C) and oxygen (O) peaks from PMMA and acrylic-based PSA samples indicates that they are acrylic-based materials, whereas Si peaks from the PDMS and silicon-based PSA samples confirm their silicon-rich nature.

Acrylic-based PSA tape and silicon-based PSA tape were used as an intermediate layer to create PDMS–PMMA constructs. These adhesives were chosen as they are considered to be biocompatible, thereby removing concerns about direct contact with materials inside the microchannels. Figure 2 shows energy-dispersive X-ray (EDX) spectra for the elemental composition of PMMA, PDMS and the two types of PSA tape. The results showed that the acrylic-based PSA and PMMA were mainly composed of carbon (C) and oxygen (O), confirming their acrylic-rich nature, whereas the presence of silicon (Si) peaks from the PDMS and silicon-based PSA samples confirmed that they were silicon-rich.

PSA is a permanently tacky tape that adheres to PMMA substrates with only light pressure (no more than hand pressure), whereas it does not readily adhere to PDMS. PMMA has moderate surface energy (41.1 mN m^−1^)^49^, and thus, has good wettability compared to PDMS which exhibits low surface energy (20.4 mN m^−1^)^50^. Substrates with higher surface energy or higher wettability exhibit a greater tendency to bond compared to those with lower energy^51^. Water contact angles were measured for untreated and oxygen plasma-treated PMMA, PDMS and PSA (Table 2). The results showed that untreated surfaces had a greater contact angle than those that had been treated, demonstrating that oxygen plasma treatment increased the surface energy of the substrates, leading to an increase in the wettability or hydrophilicity of the surfaces. As shown in Table 2, contact angle values for untreated PMMA (θ = 71.58°) and PDMS (θ = 109.84°) were in good agreement with literature reports (θ_PMMA_ ~ 68°–76°, θ_PDMS_ ~ 90°–110°)^26,52–55^, and displayed a relatively hydrophilic surface for PMMA in comparison with PDMS. In fact, PMMA showed greater wettability compared to PDMS, thereby leading to better adhesion to PSA. ‌Given that an adhesive layer needs to have a lower (or equivalent) surface energy than the corresponding substrate^40,51^, it was confirmed that PSA has no difficulty wetting PMMA, thereby explaining the observed adhesion to PMMA substrates. By contrast, PSA could not wet PDMS due to the higher surface energy of PSA compared to PDMS, thus explaining the poor bonding between these two materials. In an attempt to improve bonding, both PDMS and PSA were treated with oxygen plasma. The resulting contact angles after oxygen plasma treatment indicated a dramatic decrease for PSA and PDMS surfaces compared to the untreated cases. The increased wettability of the PDMS and PSA surfaces following the oxygen plasma treatment was a result of increased surface energy, and led to enhanced bonding. Indeed, during oxygen plasma treatment, excited plasma ions collide with a substrate and change its surface properties through the reorientation of the polar functional groups from the bulk to the surface. Ultimately, the presence of these reoriented polar groups on the oxygen plasma-treated surfaces results in greater hydrophilicity, which is likely the primary reason for the enhanced adhesion that was observed. PDMS is a silicon-rich material, and the generation of functionalized silanol groups (SiOH) after oxygen plasma treatment increases surface energy, and has been shown to promote irreversible adhesion between two PDMS substrates through the formation of ionic Si–O–Si bonds^27,30,35^. Accordingly, the probable mechanism underlying the strong adhesion between PSA and PDMS observed in the current study would be the formation of such chemical links between the polar functional groups on the surface of each of these two materials which resulted from oxygen plasma treatment.

**Table 2:** Water contact angles (θ) measured for oxygen plasma-treated and untreated surfaces.

Material	θ before oxygen plasma		θ after oxygen plasma (exposure time = 60 s)	
Silicon-based PSA		87.22°		6.86°
Acrylic-based PSA		93.95°		7.05°
PDMS		109.84°		9.71°
PMMA		71.58°		55.51°

According to the Young’s equation (γs = γsl + γl cos θ), the contact angle of a liquid drop on a solid surface (θ) is defined by the action of liquid surface tension (γl), solid–liquid interfacial tension (γsl), and solid surface tension (γs) (which is surface free energy of the solid)^51^.

### Bonding strength analysis based on delamination and tensile tests

Several methods have been reported for the creation of a permanent bond between PDMS and plastics, as demonstrated by the rupture of the PDMS surface when attempts were made to pull apart the two substrates^26,29,30,32,33^. Most of these studies have not reported the tensile strength of the PDMS–PMMA bond (see Table 1). The few studies that include PDMS–PMMA constructs have reported tensile strength values of less than 56 psi when the bonding process took place at room temperature (see the references in Table 1). In the current work, the bonding strength of the PDMS–PMMA constructs, in which flat PMMA and PDMS substrates were bonded using our new bonding method, was evaluated via delamination and tensile tests.

**Figure 3:**
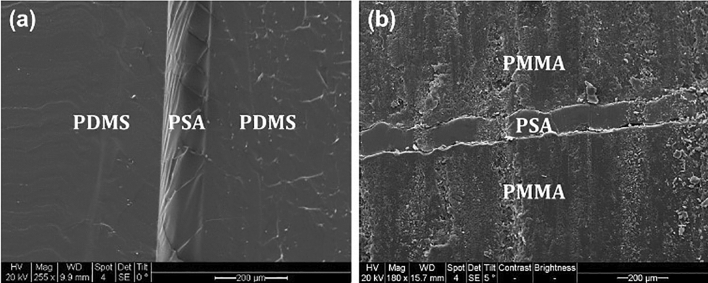
Cross-sectional scanning electron microscopy images of the (**a**) PDMS–PSA–PDMS construct where PSA is plasma bonded to PDMS and (**b**) PMMA–PSA–PMMA.

Manual delamination tests were performed on individual PMMA–PSA and PDMS–PSA constructs that had been created either with or without oxygen plasma treatment. The results consistently showed strong adhesion between PSA (both acrylic and silicon-based ones) and PMMA regardless of plasma treatment, with no residue observed when they were separated during the delamination test. In contrast, PSA simply peeled off from PDMS in the absence of oxygen plasma treatment, meaning that no appreciable bonding was realized between PDMS and PSA. However, in the presence of plasma treatment, the resulting bond between PDMS and PSA was stronger than that observed between PMMA and PSA, as evidenced by the tearing of the PDMS (as opposed to the two layers cleanly separating with no residue) during the delamination test. Examination of the cross section of the PMMA–PSA and PDMS–PSA bonds using a scanning electron microscope (SEM) showed a more desirable adhesion pattern between PSA (silicon-based) and PDMS (Fig. 3a) compared to the PMMA–PSA bond (Fig. 3b).

**Figure 4:**
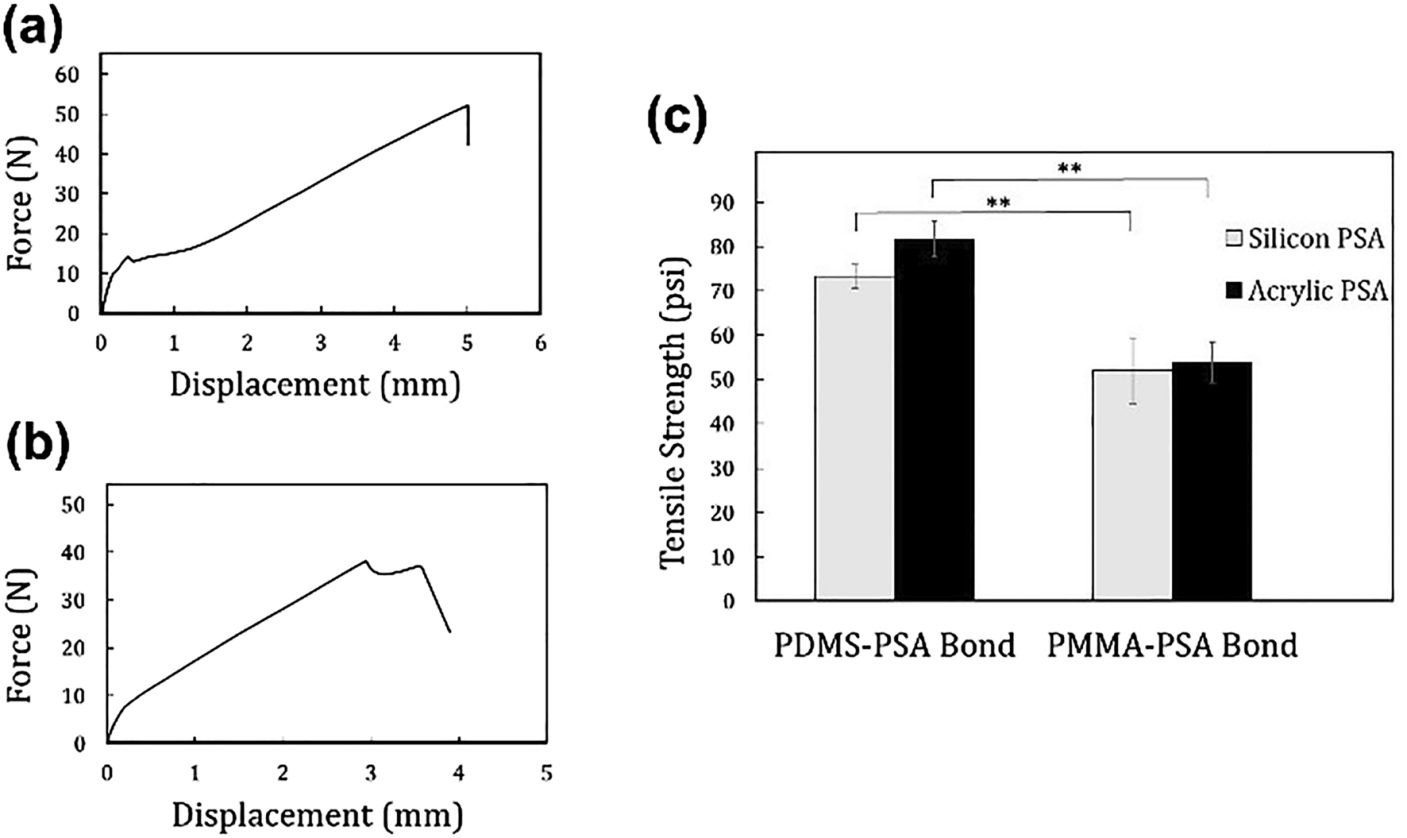
Tensile strength test of the PDMS–PSA–PMMA bond measured at a displacement rate of 3 mm min^−1^. The load–displacement curve of (**a**) PMMA–PSA and (**b**) PDMS–PSA bonds in which the PSA was silicon-based. (**c**) The average tensile bonding strength of PMMA–PSA and PDMS–PSA bonds for both acrylic and silicon-based PSAs. Tensile strength of the bond between PDMS and PSA (both acrylic and silicon PSA groups) was significantly higher than that of PMMA and PSA (p-value = 0.008 for Silicon PSA group, and p-value = 0.005 for Acrylic PSA group).

The strengths of the PMMA–PSA and PDMS–PSA bonds were quantified using a tensile strength test, in which the bonded constructs were pulled apart at a displacement rate of 3 mm min^−1^ until bond failure occurred. Since the conducted delamination tests confirmed no appreciable bonding between PDMS and PSA in the absence of oxygen plasma treatment, the tensile strength test was not conducted for constructs created without the use of oxygen plasma. Figure 4a,b are load–displacement curves for the PMMA–PSA and PDMS–PSA constructs, respectively, where the PSA was silicon-based. It is evident that the PDMS–PSA construct could withstand a higher maximum tensile force (UTS) while being pulled apart. The tensile strength of each of these constructs was also compared (Fig. 4c) using silicon-based PSA and acrylic-based PSA. The average tensile strength of the PDMS–PSA bonds (~ 82 psi for PDMS-acrylic PSA bond and ~ 73 psi for PDMS-silicon PSA bond) were significantly higher than that of PMMA–PSA bonds (~ 54 psi for PMMA-acrylic PSA bond and ~ 52 psi for PMMA-silicon PSA bond). The observed failure of the PDMS–PSA bond for both types of PSA tapes was consistently at the interface between the adhesive and its carrier while PDMS remained adhered to the adhesive, indicating a very strong bond between PSA and PDMS. In contrast, for the PMMA–PSA bond, the failure was at the interface between the adhesive and PMMA without any damage to the adhesive-carrier bond.

Considering that PSA has been reported to be a reliable adhesive to bond PMMA substrates for microfluidic applications^38,39,43^, the high level of adhesion between PSA and PDMS suggests that the new method of bonding being described here would have utility in creating PDMS–PMMA hybrid devices with PSA at the interface. The tensile strength of such constructs would be comparable, or even greater than that obtained by other reported room-temperature methods (see Table 1)^26,34^. For example, Tang et al*.*^26^ obtained 26 psi (~ 180 kPa) tensile strength in PDMS–PMMA constructs mediated by the formation of a chemically robust amine–epoxy bond at the interface of PMMA and PDMS. In another method that bonded two PMMA substrates using a thin PDMS intermediate layer, the bond strength was reported to be about 2 psi (~ 15 kPa)^27^.

### Bonding strength analysis based on clogging and leakage tests

**Figure 5:**
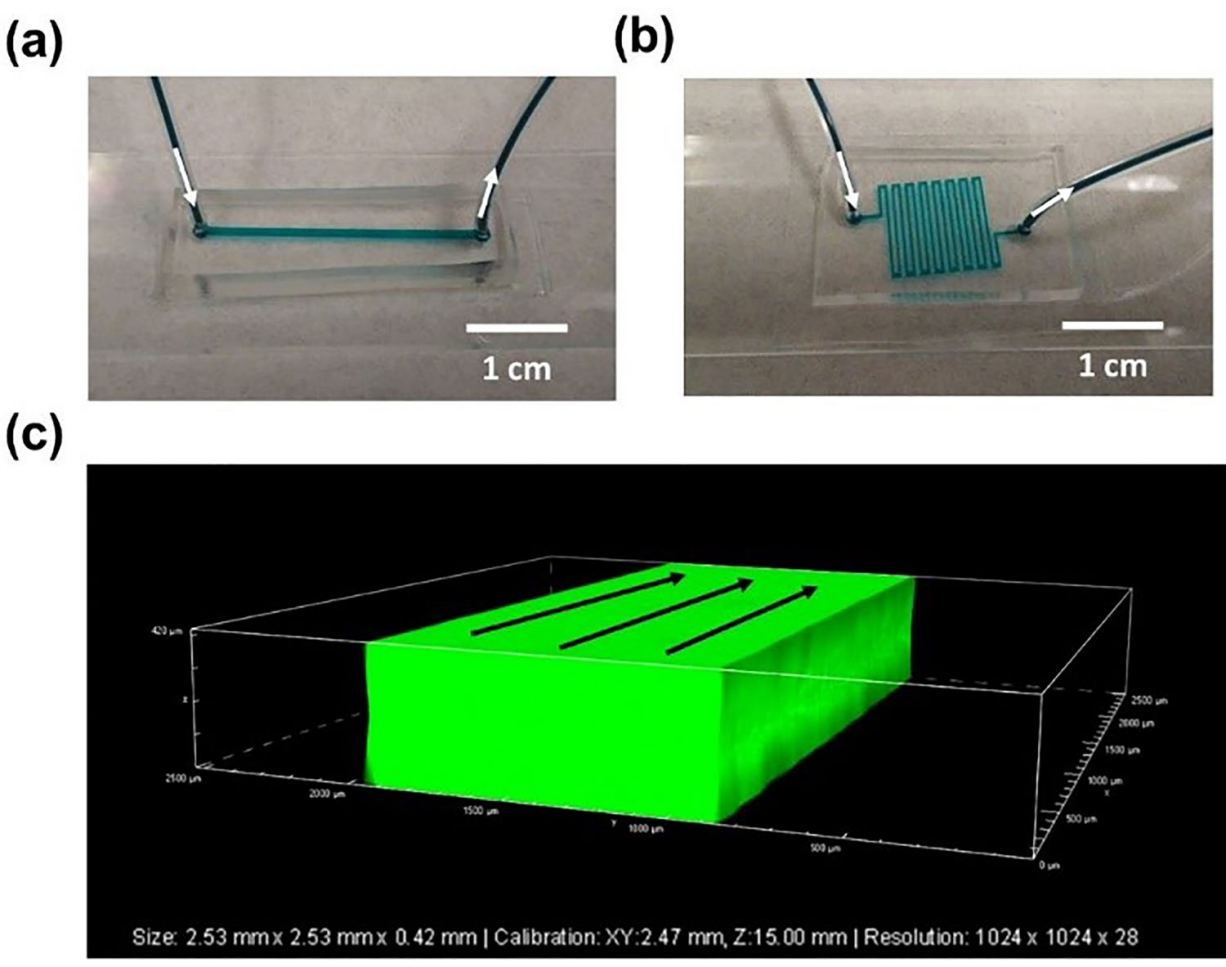
Clogging test of the fabricated microchannels. A dye solution was introduced into the (**a**) straight and (**b**) serpentine microchannels. The dye flowed through the microchannels, confirming both microchannels were totally unclogged. White arrows indicate the flow direction. (**c**) Confocal laser scanning microscopy of the straight microchannel filled with a fluorescent dye. Black arrows indicate the flow direction.

Microchannel clogging is a known issue when bonding together different materials to generate a hybrid microfluidic device. To evaluate if this was a concern for the PSA-based method described here, two types of microfluidic chips were fabricated using the newly developed bonding protocol (using both types of PSA tape)—one with a straight flow channel, and a second with a serpentine flow channel as described in the “Methods” section (see Fig. 1b). A dye solution was introduced into the inlet of a microchip, and the flow of the fluid followed through the device. As indicated in Fig. 5a,b, the microchannels were totally unclogged for both the straight and serpentine microchannel configurations, respectively. Three-dimensional (3D) visualization using confocal microscopy (Fig. 5c) confirmed that the microchannels remained totally unclogged and did not experience any wall collapse.

Leakage is a major concern for hybrid microfluidic devices. To determine if devices fabricated using PSA-based bonding of PDMS and PMMA would be prone to leakage, a dye solution was pumped into hybrid devices housing straight and serpentine microchannels at rates of 5–30 mL min^−1^. The microfluidic constructs remained sealed even at the maximum applied flow rate (30 mL min^−1^), which, on a per-minute basis, represents over 2000 times the total internal volume of the microfluidic network. This result was greater than the maximum leakage resistance reported for other hybrid microfluidic devices (10 mL min^−1^)^56^, and revealed that the PDMS–PSA bond integrity was strong enough for applications involving high flow rates or pressures, such as multiphase flow analyses in micromodels for subsurface studies^57,58^, where leakage resistance is particularly important. Furthermore, to examine the hydrolytic stability of the PDMS–PSA bond, the fabricated microfluidic devices were filled with deionized water and then immersed in water at room temperature for three weeks. Clogging/leakage tests carried out after three weeks of immersion showed no leakage even at 30 mL min^−1^, indicating the bond was resistant to hydrolytic degradation for an extended period.

### Bonding strength analysis based on a burst pressure test

**Figure 6:**
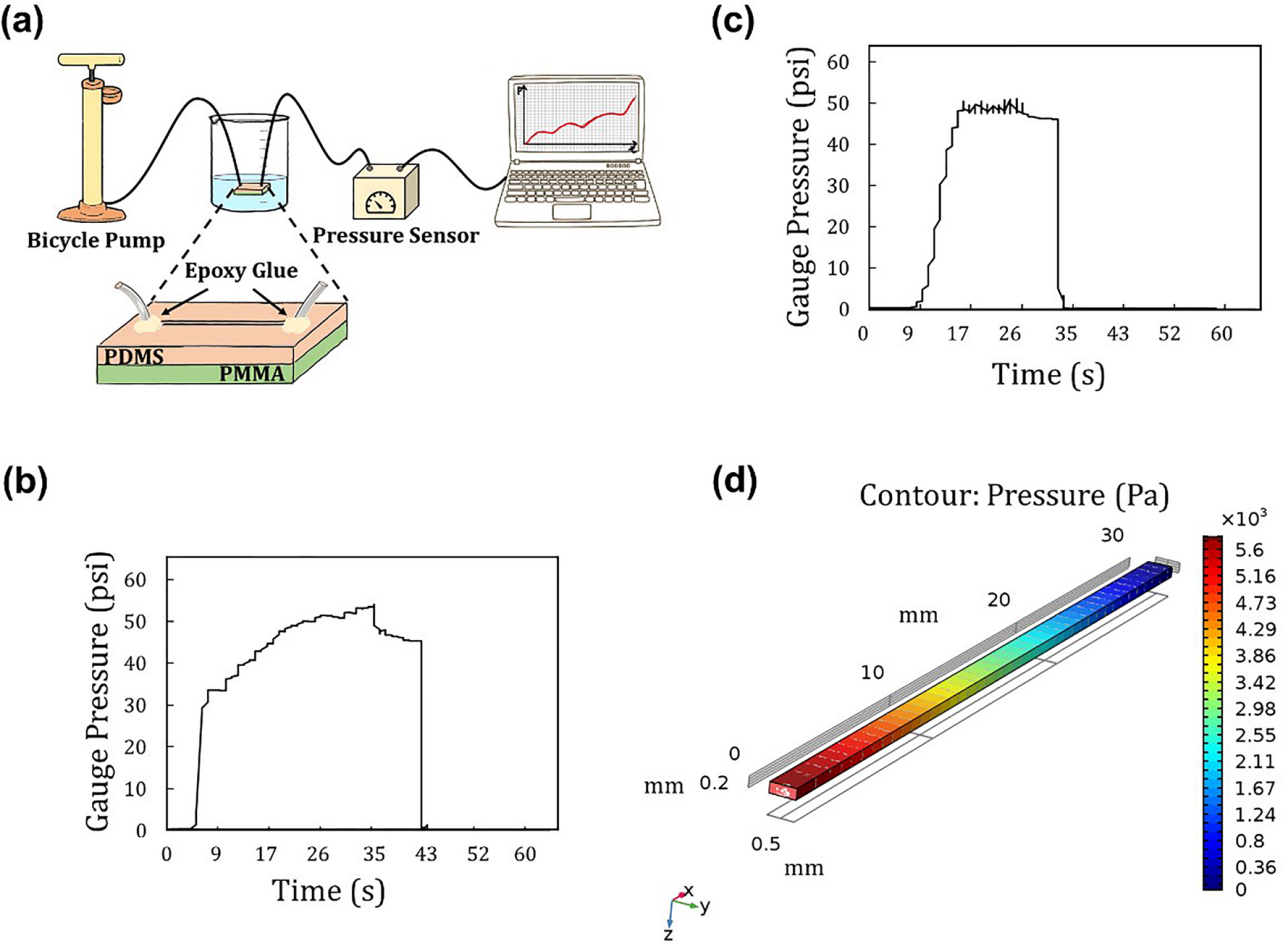
Burst pressure test of PDMS–PSA–PMMA bond (experimental set-up and results) and COMSOL simulation of leakage test. (**a**) The experimental set-up for a burst pressure test. Burst pressure test result is shown in (**b**) straight and (**c**) serpentine microchannels. The PDMS–PSA bond endured a pressure of approximately 50 psi while the pressure dramatically dropped not due to the bond failure, but rather a connection leak close to the air pump. (**d**) Simulation of pressure distribution in the straight microchannel, modeled by COMSOL at a flow rate of 30 mL min^−1^ for dye solution.

The strength of the PDMS–PSA bond was further investigated using a burst pressure test in which air was injected into a dead-end microchannel to measure the failure pressure of the bonded construct. Figure 6a schematically shows the set-up of the experiment. Since the previously conducted tensile tests showed no significant difference between acrylic and silicon-based PSA groups, the burst pressure was measured for only the silicon-based PSA group. Figure 6b,c display the pressure graphs for the straight and serpentine microchannels, respectively. According to the data collected, the pressure increased as a function of time, and then underwent a sudden and dramatic drop. Whereas such a drop is indicative of bond failure, the likely reason for the drop in this case was a leak that initiated at the pump connection, despite being sealed with Teflon tape and epoxy glue. Despite this leak, this test showed that all the bonded PDMS–PSA constructs evaluated successfully endured a minimum pressure of 50 psi (~ 345 kPa). Given that microchannels that are considered to be irreversibly sealed can withstand a pressure of 30–50 psi^59^, the obtained burst pressure of 50 psi for the bonded PDMS–PSA constructs in the current study supports an irreversible bond between PSA and PDMS after oxygen plasma treatment. It should be noted that the burst pressure measured here was significantly higher than the pressure produced in a microchannel at a flow rate of 30 mL min^−1^ during the previously described leakage test. Figure 6d presents the fluid pressure distribution in a straight microchannel at a flow rate of 30 mL min^−1^ as simulated using COMSOL. Considering that the pressure inside most microfluidic devices has been reported as being approximately 15 psi (~ 100 kPa)^60^, the obtained burst pressure of 50 psi for the PDMS–PSA bond under consideration here is clearly high enough for many microfluidic applications, including high pressure liquid chromatographic separation or high pressure centrifugal microfluidic assays.

## Conclusions and future work

There are many microfluidic applications which can benefit from a hybrid device made up of PMMA and PDMS. However, current methods to generate such a device are inefficient, cumbersome and require high temperatures. In this study, a simple, rapid, efficient, and low-cost method was described to bond PMMA and PDMS at room temperature using commercially available, double-sided PSA tape at the interface. The key step in the process was oxygen plasma treatment, which enabled PSA and PDMS layers to form an irreversible bond but did not cause microchannel clogging or deformation. The bond was hydrolytically stable and tolerated relatively high fluid flow rates and pressures without loss of integrity. Bond strength was deemed to be high through tensile and burst pressure tests. The simplicity of this new bonding approach will promote the creation of PDMS-based microfluidic devices in makerspaces, and will support the rapid production of hybrid PDMS–PMMA microfluidic devices for a wide range of biomedical applications such as centrifugal microfluidic assays, liquid chromatographic separation, microvalve/micropump fabrication and gas-controlled cell chemostats.

## Methods

### Materials and reagents

PDMS base polymer and curing agent were purchased from Dow Corning Corp (Sylgard 184). PMMA (1.5 mm thick) and PSA (ARseal 90880 and ARcare 8939) sheets were purchased from McMaster-Carr (USA) and Research Adhesive (USA), respectively. Tygon Microbore tubing (outside diameter of 1.52 mm, inside diameter of 0.51 mm) and fluorescent dye (Kingscote-Mfr#506250-RF4) were purchased from Cole-Parmer (Canada).

### Microchannel fabrication

Two commonly used microchannel geometries (straight and serpentine) were fabricated on PDMS using standard photolithography technique^4,61,62^. Figure 1b shows schematics of these microchannels which were used to analyse bonding strength under fluid flow conditions. Microchannels were designed using computer-aided design (CAD) software (AutoCAD, Autodesk), and then the pattern was transferred onto a high-resolution transparency sheet as a mask. A SU-8 master mold was fabricated using a SU-8 photoresist patterned on a silicon wafer. The patterning includes spin coating of the SU-8 photoresist on silicon wafer, followed by soft baking, UV exposure through the mask, hard baking, and chemical development. Following the fabrication of the master mold, PDMS base polymer and curing agent were mixed at the ratio of 10:1, and air bubbles were removed under vacuum. The mixture was poured onto the mold and cured in an oven at 120 ℃ for 20 min. The generated PDMS sheet (patterned with a microchannel) was peeled off from the mold, and two holes were punched in the sheet at the inlet and outlet ports of the microchannel. To create a hybrid device, the fabricated microchannel patterned PDMS layer was bonded to a flat PMMA layer using PSA as an intermediate layer. The PMMA and PSA used had been previously cut into the appropriate dimensions using a laser cutter (TEN-HIGH CO2, 40 W, 110 V/220 V).

### Energy-dispersive X-ray (EDX) analysis

EDX spectroscopy was performed to characterize PMMA and PDS, and specifically to identify the elemental composition of the PSA tapes. This test was used to investigate whether the observed bonding between PDMS and PSA substrates was dependent on the PSA composition. EDX analysis of PDMS, PMMA and PSA substrates was performed using a Rayny EDX-720 (Shimadzu, Kyoto, Japan) operated at an accelerating voltage of 15–20 kV.

### Contact angle measurement

Water contact angle measurements were performed to evaluate the wettability and surface energy of the substrates before and after oxygen plasma treatment. A contact angle goniometer (Ramé-Hart Instruments Co., 500-F4) was used to measure the contact angle of a liquid droplet against a substrate, thereby determining the tendency of the substrate toward bonding. Substrates with higher surface energy exhibit higher wettability or hydrophilicity, and thus show smaller contact angles^51^.

### Scanning electron microscopy (SEM) examination

Using SEM, cross sections of the bonded constructs were examined to visually assess adhesion quality between PSA and PMMA, and PSA and PDMS. Flat PDMS and PMMA substrates were used to prepare PDMS–PSA–PDMS and PMMA–PSA–PMMA bonded constructs. The constructs were then cut cross-sectionally and imaged using Quanta SEM (FEI Company, Quanta 250 FEG).

### Confocal laser scanning microscopy (CLSM) examination

CLSM was used to examine whether the microchannel structures collapse or become clogged during the process of bonding. A microchannel-patterned PDMS layer was bonded to flat PMMA using PSA as an intermediate layer, and then the sealed chip was filled with a fluorescent dye (Kingscote-Mfr #506250-RF4). The dye was subsequently visualized at 488 nm using a confocal microscope (Nikon AR2).

### Delamination and tensile tests

To investigate bond strength, delamination and tensile tests were performed on PMMA–PSA and PDMS–PSA constructs in which PSA was bonded to flat PMMA or PDMS substrates. For PMMA–PSA constructs, PSA was laminated on PMMA and the entire construct was compressed via a trigger clamp for 2 min. For PDMS–PSA constructs, both substrates were treated with oxygen plasma and then compressed slightly against each other for 10–15 s. The delamination test was conducted manually by simply peeling off the bonded PSA. The bonding strength was also quantitatively evaluated using a tensile strength tester (ElectroForce 3220-AT Series II), in which the bonded constructs (with a total bonded surface area of 1 cm^2^) were pulled apart at a displacement rate of 3 mm min^−1^ until the bond failed. The tensile test measured the ultimate tensile strength (UTS), reported at the highest load reading that occurred at the point of bonding failure.

### Leakage test

Leakage of the PDMS–PMMA hybrid devices was tested by injecting dye solution into the microchannels. The flow rate of the dye solution was started at 5 mL min^−1^ and increased up to 30 mL min^−1^ by increasing the injection rate by 5 mL min^−1^. A syringe pump (Harvard Apparatus, PHD 2000 Infusion) was used to inject the dye and control its flow rate. These tests were performed on two types of fabricated microchannels (Fig. 1b)—one was a straight channel (total length of 30 mm, height of 0.4 mm, and width of 1 mm), and another was a serpentine channel (total length of 190 mm, height of 0.4 mm, width of 0.3 mm).

### Burst pressure test

To measure the maximum pressure that hybrid devices are able to withstand prior to bursting, air was manually injected inside the microchannels using a bicycle pump while the outlet was blocked. The pressure within the channels was recorded by a micro-gauge pressure sensor (Honeywell, model SSCDANN150PG2A3) until the bond failed. To prevent air leaks in the connections, they all were wrapped with Teflon tape and glued with epoxy. The burst pressure tests were performed on both the straight and serpentine microchannels.

### Statistical analysis

All experiments were repeated independently three times and data were presented as mean ± standard error. Differences between groups were analyzed by student's t-test where a probability value below 0.05 was considered statistically significant.
